# High-Frequency Optical Coherence Tomography: Addressing Common Pitfalls of Traditional Optical Coherence Tomography Imaging

**DOI:** 10.1016/j.jscai.2025.102621

**Published:** 2025-03-18

**Authors:** Brian C. Case, Ryan Wallace, Daniel Chamié

**Affiliations:** a Section of Interventional Cardiology, MedStar Washington Hospital Center, Washington, DC; b Department of Cardiology, MedStar Washington Hospital Center, Washington, DC; c Section of Cardiovascular Medicine, Department of Internal Medicine, Yale School of Medicine, New Haven, Connecticut

**Keywords:** high-frequency optical coherence tomography, intravascular imaging, percutaneous coronary intervention

Current guidelines support the use of intravascular imaging (IVI) during percutaneous coronary intervention (PCI), given the association with reduced ischemic events, including all-cause and cardiovascular mortality.^1^, ^2^, ^3^, ^4^, ^5^ Despite this recommendation, rates of IVI use for PCI guidance have only slightly risen, and overall usage remains low (∼17%).^6^

The slow rate of adoption of IVI, particularly optical coherence tomography (OCT), has often been attributed to multiple weaknesses of current-generation devices. The need for contrast injection to clear the vascular lumen from blood during image acquisition adds complexity to obtaining images of adequate quality and increases the total procedural contrast volume. Concerns about contrast volume are usually referred to as a limitation of OCT use in patients with kidney dysfunction, multivessel disease, or when several imaging runs are anticipated during complex interventions. Current-generation OCT devices have a limited pullback length of 75 mm, making them less optimal in cases of long diffuse lesions, for assessing long segments of overlapping stents, or in myocardial infarction with no obstructive disease, where scanning a long vessel segment is needed to identify the culprit lesion. In these situations, >1 imaging pullback is usually necessary for a complete vessel assessment, adding time and contrast to the procedure. Furthermore, the profile of current-generation OCT catheters limits the evaluation of severe stenoses, as the imaging catheter obstructs the lumen at the minimal lumen area (MLA) site, preventing contrast flushing and visualization of the vessel distal to the stenosis.

In this issue of *JSCAI*, Quimby et al^7^ describe a multicenter experience with a newer generation high-frequency OCT (HF-OCT) imaging system (Gentuity LLC) in a variety of clinical scenarios. HF-OCT addresses some of the shortcomings of previous-generation OCT catheters. The reduction in catheter size from 2.7F to 1.8F facilitates easier crossing and evaluation of tight lesions. Compared with the current-generation OCT, HF-OCT features a longer (100 mm) and faster (1 second) pullback, reducing the need for repeat pullbacks and minimizing contrast use.^8^ Despite its lower profile, the HF-OCT catheter provides a larger field of view, allowing visualization of vessels up to 14 mm in diameter.

The authors analyzed 143 HF-OCT pullbacks in 81 unique coronaries from 75 patients. The primary end point was the pullback length with clear images, defined as visualization of the vessel wall along an arc of at least 270°. The proximal and distal reference lumen areas ranged from 3.2 to 27.6 mm^2^ and 1.2 to 14.6 mm^2^, respectively, reflecting the inclusion of a wide range of vessel sizes; left main coronary arteries were imaged in 74 (51.7%) of the HF-OCT acquisitions. The mean clear image length was 68.4 mm and was not significantly different whether analyzed pre-PCI (67.0 mm) or post-PCI (69.4 mm). Of interest is the analysis of the 12 vessels with an MLA ≤0.63 mm^2^, the nominal area occupied by the current-generation 2.7F catheter (0.9 mm), beyond which the imaging catheter would theoretically obstruct contrast flushing and visualization of the distal vessel. Remarkably, HF-OCT provided clear images in 83.3% of the segments distal to this very tight MLA without lesion predilatation. This advantage is appealing to evaluate the coronary lesion morphology before any lesion manipulation, to identify the mechanism or the culprit lesion for an acute coronary syndrome or the mechanisms of stent failure. This benefit is less certain when the decision to proceed with PCI has been made. During PCI of tight lesions, the current practice is to image after lesion predilatation and administration of intracoronary nitroglycerin. An imaging pullback after gentle predilatation still allows for identification of high-risk plaque characteristics that require advanced preparation techniques and enable better assessment of the distal reference size.^9^ Whether the ability to image across tight lesions with HF-OCT would change this practice and simplify the PCI workflow needs to be further explored.

OCT is also often avoided in large vessels, especially in cases of left main disease, due to the limited field of view and concerns related to suboptimal blood clearance in large lumens, none of which affected the performance of HF-OCT in the current study.

We expected that imaging longer vessel segments in just 1 second would reduce the contrast volume dispensed per OCT acquisition; however, the authors reported acquiring HF-OCT runs with hand injection of 10 mL of contrast (65.7% of cases) or 10 to 14 mL dispensed by an automatic injector set at 4 mL/s (34.3% of cases)—parameters that reflect the practice with current-generation OCT systems. This contrasts with a small, single-center experience in which an average of 5.0 mL of contrast was used per HF-OCT imaging of 28 vessels.^8^ Adjustments in the best clinical practices need to follow technological advancements to optimize outcomes. Additionally, the feasibility of acquiring good-quality HF-OCT images by flushing the vessel with saline solution as a contrast substitute must be explored. Whether the combination of a faster pullback and less contrast use will impact the decision to use OCT more frequently in complex and multivessel disease PCI also needs to be determined.

In the search for maximal efficiency, the Vis-Rx catheter (Gentuity LLC) connects to a patient interface module attached to the bed rail, eliminating the need to bag an extra piece of motorized pullback device that usually lays on top of the sterile field.

Lastly, in line with the most contemporary IVI systems, HF-OCT employs artificial intelligence for real-time lumen segmentation, with fully automated identification of lumen areas, MLA, and quantification of the severity of lumen stenosis. Artificial intelligence on the HF-OCT also detects the stented region, automatically calculates stent expansion, and identifies and eliminates the presence of the guide catheter from the analysis, ultimately removing sources of confusion and optimizing the workflow of image quantification and interpretation.

Quimby et al^7^ demonstrate that the technological advancements of HF-OCT translate into improvements in image acquisition while maintaining an excellent safety profile. We are hopeful that the downsizing and simplifying to maximize efficiency and performance will also maximize adoption of IVI during PCI and outcomes in multiple clinical scenarios for which OCT use is typically avoided or not feasible.
